# AMBAR, an Encouraging Alzheimer's Trial That Raises Questions

**DOI:** 10.3389/fneur.2020.00459

**Published:** 2020-05-29

**Authors:** David A. Loeffler

**Affiliations:** Beaumont Research Institute, Department of Neurology, Beaumont Health, Royal Oak, MI, United States

**Keywords:** Abeta, albumin, Alzheimer's, AMBAR, clinical trial, intravenous immunoglobulin, peripheral sink hypothesis, plasma exchange

## Abstract

Grifols' recent Alzheimer Management by Albumin Replacement (“AMBAR”) study investigated the effects of plasmapheresis with albumin replacement, plus intravenous immunoglobulin (IVIG) in some subjects, in patients with mild-to-moderate Alzheimer's disease (AD). AMBAR was a phase IIb trial in the United States and a phase III trial in Europe. There were three treatment groups (plasmapheresis with albumin replacement; plasmapheresis with low dose albumin and IVIG; plasmapheresis with high dose albumin and IVIG) and sham-treated controls. Disease progression in pooled treated patients was 66% less than control subjects based on ADAS-Cog scores (*p* = 0.06) and 52% less based on ADCS-ADL scores (*p* = 0.03). Moderate AD patients had 61% less progression, based on both ADAS-Cog and ADCS-ADL scores, than their sham-treated counterparts (*p*-values 0.05 and 0.002), and their CDR-Sb scores declined 53% less than their sham-treated counterparts. However, ADAS-Cog and ADCS-ADL scores were not significantly different between actively-treated and sham-treated mild AD patients, although CDR-Sb scores improved vs. baseline for treated mild AD patients. Patients administered both IVIG and albumin had less reduction in brain glucose metabolism than sham-treated patients. Questions raised by these findings include: what mechanism(s) contributed to slowing of disease progression? Is this approach as effective in mild AD as in moderate AD? Must IVIG be included in the protocol? Does age, sex, or ApoE genotype influence treatment response? Does the protocol increase the risk for amyloid-related imaging abnormalities? How long does disease progression remain slowed post-treatment? A further study should allow this approach to be optimized.

## Introduction

The amyloid hypothesis (1) led to efforts to treat Alzheimer's disease (AD) by reducing brain Aβ, including vaccination (2), anti-Aβ antibodies (3–7), Aβ aggregation inhibitors (8), β-secretase inhibitors (9), and γ-secretase modulators (10), and inhibitors (11). The failure of these approaches to slow AD's progression [with the possible exception of anti-Aβ antibody Aducanumab, whose recently released findings are controversial (12)] resulted in increased targeting of tau, the main component of neurofibrillary tangles (NFTs), by vaccination (13), anti-tau antibodies (14), tau aggregation inhibitors (15), and kinase inhibitors (16). Other mechanisms which may contribute to AD's neuropathology including inflammation (17), oxidative stress (18), and excitotoxicity (19) have also been targeted, with negative results except for the N-methyl-d-aspartate receptor antagonist Memantine HCl. Memantine and cholinesterase inhibitors are the only treatments currently approved by the United States Food and Drug Administration for AD; they provide symptomatic benefits to some patients but are not disease modifiers (20).

This Perspective will discuss the results, significance, possible mechanisms, and questions raised by the recently-completed Alzheimer Management by Albumin Replacement (“AMBAR”) study (ClinicalTrials.gov ID: NCT01561053) (21) performed by Grifols (Instituto Grifols, S.A.). AMBAR was registered as a phase IIb study in the United States and a phase III study in Europe. The protocol involved plasma removal and its replacement with therapeutic-grade human albumin, plus supplementation with intravenous immunoglobulin (IVIG) in some patients. The rationale for the study was that lowering plasma Aβ levels by this approach might reduce brain levels of soluble Aβ, as predicted by the “peripheral sink hypothesis” (22, 23), possibly slowing AD's progression.

## Background

*In vitro* studies found that albumin inhibited Aβ aggregation (24) and neurotoxicity (25). However, plasma albumin from AD patients is more glycated and nitrotyrosinated than plasma from healthy subjects, reducing its ability to inhibit Aβ aggregation (26). Grifols theorized that replacing AD patients' albumin with therapeutic-grade albumin should overcome this problem. Further, therapeutic-grade albumin should more effectively bind plasma Aβ and sequester it than plasma albumin from AD patients. Albumin may protect neurons by additional mechanisms, including anti-oxidant (27, 28) and anti-inflammatory (29, 30) activities. Because of albumin's anti-Aβ effects, Grifols decided to explore the potential of its human plasma albumin Albutein® (31) for treating AD.

The peripheral sink hypothesis is based on the finding that administration of a monoclonal anti-Aβ antibody to a transgenic mouse AD model lowered brain Aβ, despite apparent failure of the antibody to enter the brain (22, 23). This suggested that lowering plasma albumin might result in reduction of brain Aβ by increasing movement of soluble Aβ from brain into peripheral blood. The hypothesis assumes that soluble Aβ is in equilibrium between brain and peripheral blood. Grifols theorized that because ~90% of plasma Aβ is bound to albumin (32), replacing AD patients' plasma with Albutein, which does not contain detectable Aβ (33), should decrease plasma Aβ (34). The hypothesis predicted that this would result in increased movement of soluble Aβ out of the brain. Some studies have supported the peripheral sink hypothesis (35–37) but others have not (38–40).

## Preliminary Studies

In 2005 Grifols performed a pilot study (41) with seven mild-to-moderate AD patients who underwent plasma removal with Albutein replacement twice weekly for 3 weeks with a 6-months follow-up period. No clear patterns were detected for changes in plasma Aβ40 or Aβ42. CSF Aβ40 decreased slightly during plasma exchange with a greater decrease in CSF Aβ42, and both Aβ concentrations returned to near baseline 6 months post-treatment. Mini-Mental State Examination (MMSE) and Alzheimer's Disease Assessment Scale–Cognitive subscale (ADAS-Cog) scores changed little, while imaging suggested increased hippocampal volume and increased frontal and temporal cortex perfusion. In a 1-year extension of the study, a more sensitive method for measuring plasma Aβ40 and Aβ42 revealed a “sawtooth” pattern: Aβ decreased after each plasma exchange, and returned to baseline before the next procedure. CSF Aβ40 and Aβ42 remained relatively stable during the extension. Grifols concluded from these findings that the approach was feasible to consider for treatment of AD patients.

In 2007 Grifols performed a phase II trial (ClinicalTrials.gov Identifier: NCT00742417) (42, 43) with this approach, involving 19 actively-treated and 20 sham-treated mild-to-moderate AD patients. The treatment group underwent plasma removal with Albutein replacement twice weekly for 3 weeks, then weekly for 6 weeks followed by every 2 weeks for 12 weeks. Control patients underwent simulated procedures so neither patients nor study raters knew patient group assignments. Parameters measured were similar to those in the pilot study, following patients for 6 months. The adjusted (least-squares) mean CSF Aβ42 concentration was “marginally higher” (*p* = 0.07), in the treatment group compared to the control group, after the last plasma exchange compared to the mean baseline value, while the change from baseline in CSF Aβ40 was not significantly different between groups. A sawtooth pattern for plasma Aβ40 and Aβ42 was again found in the treatment group. MMSE and ADAS-Cog scores tended to be higher in the treatment group than in the control group at the end of treatment and follow-up periods but between-group differences were not significant (ADAS-Cog *p* = 0.09 at week 21, MMSE *p* = 0.08 at week 44). Higher scores in the treatment group were found for some tests of language and attention, but worse scores for the Neuropsychiatric Inventory (NPI) (44). The frequency of adverse events was similar between groups.

## Ambar

AMBAR was a multicenter, randomized, double-blind, placebo-controlled study in which patients were treated for 14 months. The study included 496 patients with mild to moderate AD (MMSE scores 18–26), divided among three groups of actively-treated subjects and a sham-treated control group. All actively-treated patients initially underwent removal of 2,500–3,000 mL of plasma (“high-volume” plasma exchange), replaced by the same volume of Albutein 5%, weekly for 6 weeks through a peripheral vein or a central venous catheter placed in the subclavian or jugular vein. This was followed by 12 months of monthly “low-volume” plasma exchange in which 650–880 mL of plasma was removed and replaced with 100 mL of Albutein 20% (20 g Albutein), 100 mL of Albutein 20% plus 200 mL of Grifols' IVIG Flebogamma 5% DIF (10 g Flebogamma) (“low albumin/low IVIG” group), or 200 mL of Albutein 20% (40 g Albutein) plus 400 mL of Flebogamma 5% DIF (20 g Flebogamma) (“high albumin/high IVIG” group). This second stage of plasmapheresis was performed via a peripheral vein. ADAS-Cog and Alzheimer's Disease Cooperative Study-Activities of Daily Living (ADCS-ADL) scores were measured at baseline, after initial plasmapheresis, at 7, 9, and 12 months of second stage plasmapheresis, and at 14 months after finishing plasmapheresis. Primary outcome measures were changes in ADAS-Cog and ADCS-ADL scores between baseline and endpoint. Secondary outcome measures were changes in cognitive, functional, and behavioral tests, measures of disease progression, and alterations in CSF p-tau, total tau, Aβ40, and Aβ42, plasma Aβ40 and Aβ42, brain structure, and brain glucose metabolism. Statistical analyses of changes vs. sham-treated controls in ADAS-Cog and ADCS-ADL scores were performed on data from pooled treatment subjects and, in pre-specified analyses, from patients with mild AD (MMSE 22–26) and moderate AD (MMSE 18–21).

AMBAR's topline results (45) indicated that treatment groups averaged 50 to 75% less worsening of ADAS-Cog scores and 42 to 70% less worsening of ADCS-ADL scores than control subjects. Pooled data from treated subjects showed that these patients declined, on average, 66% less than control subjects based on ADAS-Cog scores (*p* = 0.06) and 52% less based on ADCS-ADL scores (*p* = 0.03). Analyses of changes from baseline to endpoint in patients with moderate AD found 61% less disease progression, based on both ADAS-Cog and ADCS-ADL scores, than sham-treated moderate AD patients (*p* = 0.05 for ADAS-Cog, 0.002 for ADCS-ADL). Although some slowing of disease progression was also found in the treated patients with mild AD, a similar pattern was unexpectedly seen for sham-treated mild AD patients so the between-group differences in ADAS-Cog and ADCS-ADL scores were not statistically significant.

At the 2019 International Congress on Alzheimer's and Parkinson's (AD/PD) (46, 47) Grifols reported significant differences at endpoint between patients in the high albumin/high IVIG treatment arm and the control subjects in tests of memory, language, processing speed, and quality of life. Actively-treated moderate AD patients performed significantly better than their sham-treated counterparts on tests of memory and quality of life, while mild AD patients performed significantly better than their control counterparts on tests of language and processing speed. A low rate of adverse events was reported, occurring mainly during high-volume plasma exchange. CSF Aβ42 was stable in treated patients while decreasing in sham-treated patients (results for Aβ40 were not shown), while CSF phosphorylated and total tau increased less in treated patients than in controls. At the 2019 Alzheimer's Association International Conference (48) Grifols reported that Alzheimer's Disease Cooperative Study-Clinical Global Impression of Change (ADCS-CGIC) scores had remained stable in all treatment groups, and these patients had declined, on average, 71% less than controls on the Clinical Dementia Rating-Sum of Boxes (CDR-Sb) scale (49, 50). CDR-Sb scores for mild AD patients improved while moderate AD patients' scores declined 53% less than their sham-treated counterparts (51). Final results presented at the 2019 Clinical Trials on Alzheimer's Disease (CTAD) Conference indicated that patients receiving both Flebogamma and Albutein had less reduction in brain glucose metabolism than controls.

## Discussion

The results from AMBAR are encouraging, in contrast to the other approaches that have been tried to slow AD's progression. A review of AD trials for the period between 2002 and 2012 concluded that the overall success rate was 0.4% (52). No new drugs have been approved for treatment of AD since 2003, although Namzaric, which combines Memantine and Donepezil, received FDA approval in 2014.

Perhaps the most important question raised by AMBAR's findings is: what mechanism was responsible for slowing disease progression? Identifying this mechanism would provide support for further efforts to slow AD's progression by means of the same mechanism. Among the mechanisms that could have contributed to AMBAR's slowing of disease progression are reductions in neurotoxic Aβ species, tau pathology, neuroinflammation, oxidative stress, microcirculatory deficits, and neurotoxic auto-antibodies. These will be discussed below.

### Reduced Aβ

Although both Aβ40 and Aβ42 were measured in CSF, results were reported only for Aβ42 (47, 49), whose concentrations were stable in treated patients while decreasing in control patients. Whether brain levels of Aβ were lowered is unclear. CSF Aβ42 is reduced in AD (53), possibly due to sequestration of Aβ42 as insoluble fibrils (54). Lowering soluble Aβ42 in brain could either increase or decrease CSF Aβ42, depending on its rates of clearance from brain to CSF and from CSF to peripheral blood. A future study should measure CSF levels of Aβ soluble oligomers, which may be Aβ's most neurotoxic conformation (55). An assay for their measurement in CSF was recently reported (56). To determine if plaque counts were lowered, PET Aβ imaging could be performed (57, 58). Post-mortem evaluation of plaques and NFT should also be considered on subjects who pass away during a future study with the AMBAR protocol. Plaque densities are less strongly correlated than NFTs with cognitive loss in AD patients (59, 60), so even if plaque counts decreased relative to sham-treated subjects, this would be unlikely to be the sole mechanism responsible for slowing of disease progression. In the AN1792 Aβ vaccination trial, for example, despite marked reductions in plaque counts found in subsequent post-mortem studies (61, 62), clinical progression was not slowed (2). Finally, it would be worthwhile to determine the incidence of amyloid-related imaging abnormalities (ARIA). ARIA refers to imaging abnormalities (often not associated with symptoms) associated with increased movement of Aβ from brain after treatment with anti-Aβ antibodies (5, 63, 64).

### Reduced Tau Pathology

The amyloid hypothesis (1) suggests that tau pathology in AD develops downstream from Aβ deposition; therefore if the AMBAR protocol reduced brain Aβ levels, this could have secondarily decreased tau pathology. Total and phosphorylated tau (p-tau) levels in CSF are increased in AD (65). CSF levels of total and p-tau increased less in AMBAR's plasma exchange-treated patients than in sham-treated patients (47), suggesting that tau pathology may have been reduced. A future study should examine this issue by PET imaging (66). CSF concentrations of soluble tau oligomers could also be measured (67).

### Reduced Inflammation

Chronic systemic inflammation has been associated with increased risk for development (68) and progression (69) of AD. Plasmapheresis removes inflammatory cytokines from peripheral blood (70), so the AMBAR protocol could have reduced systemic inflammation via this mechanism, perhaps decreasing brain inflammation as a consequence. Inflammatory cytokines and chemokines, as well as complement proteins and activation fragments, are readily measured in CSF (71–75), so it would be useful to measure these. Activated microglia (76) and astrocytosis (77) can be imaged in the brain via PET, so these procedures should also be considered.

### Reduced Oxidative Stress

Oxidative stress in present in AD and may contribute to its pathogenesis (78). The AMBAR protocol could have directly reduced brain oxidative stress due to the anti-oxidant actions of albumin (79, 80) if CSF levels of albumin were sufficient to exert these effects. Conflicting reports have been published regarding the effects of plasmapheresis on oxidative stress (81–84). This could be examined in a future study by measuring CSF oxidative stress biomarkers such as 8-hydroxy-2'-deoxyguanosine (85), 8-isoprostane (86), protein sulfhydryls (87), and total antioxidant capacity (88).

### Reduction of Microcirculatory Deficits

Plasmapheresis with removal of low density lipoproteins is used to treat conditions such as familial hypercholesterolemia and peripheral arterial disease. This improves microcirculation and lowers systemic oxidative stress (81). AMBAR's inclusion criteria included diagnosis of AD based on NINCDS-ADRDA criteria, and imaging showing the absence of cerebrovascular disease [which includes stroke, transient ischemic attack (TIA), subarachnoid hemorrhage, and vascular dementia (89)], so AMBAR's participants likely did not have vascular dementia. However, AMBAR's exclusion criteria did not include lipid profile abnormalities, so improved microcirculation might have contributed to slowing of disease progression in some patients. Correlations between plasma lipid profile components and AD progression could be examined in Grifols' next study.

### Removal of Autoimmune Antibodies

Plasmapheresis is used to treat some autoimmune disorders because it removes pathogenic auto-antibodies as well as complement proteins and cytokines from plasma (90, 91). Some investigators have suggested that AD may be an autoimmune disorder (92, 93) although this view is not generally accepted. If autoantibodies do play a role in AD pathogenesis, then their removal may have contributed to AMBAR's slowing of AD progression, although this scenario is considered to be unlikely. In the next study with the AMBAR protocol, the presence and titers of CSF anti-hippocampal antibodies (94) could be compared in pre- and post-treatment CSF samples from both actively-treated and sham-treated subjects.

In addition to these mechanisms, plasma exchange removes many other proteins (42, 95) so the possibility is not ruled out that slowing of AD's progression could have been due to lowering of brain levels of unidentified proteins (96).

Grifols reported a low rate of adverse events in AMBAR, many of which occurred during the initial stage of plasmapheresis, which, for some patients, involved placement and 6-weeks maintenance of a central venous catheter. In the phase II trial, anxiety relating to these catheters was suggested to contribute to worse NPI scores in treated patients than in sham-treated patients (42). The decision whether to perform the initial plasma exchange through a peripheral or central vein was “based on the individual characteristics of the patient” (97). The saw-tooth pattern of plasma Aβ40 and Aβ42 was found for both the “high-volume” and “low-volume” stages of plasma exchange, so a future study should clarify if the high-volume plasma exchange (and central venous catheter) is actually necessary.

It is unclear if AMBAR's protocol is as effective in slowing disease progression in mild AD as in moderate AD; this needs clarification. Changes from baseline to endpoint in ADAS-Cog and ADCS-ADL scores indicated significant slowing of progression in the actively-treated moderate AD patients compared to sham-treated moderate AD patients, but no significant differences were found in these scores between actively-treated and sham-treated mild AD patients; however, CDR-Sb scores were improved for actively-treated vs. sham-treated mild AD patients. Although positive effects were reported for mild AD patients in tests of language and processing speed, these effects were notably absent for tests of memory.

Two of AMBAR's treatment groups included Flebogamma. Disappointing results were obtained with IVIG products in phase II and phase III AD trials (98, 99) so IVIG is no longer being considered for AD monotherapy. AMBAR's most positive results with regard to slowing of disease progression were in the high albumin/high IVIG treatment group (46), and neuroimaging similarly found that less reduction in brain glucose metabolism vs. sham-treated patients was found “particularly in patients receiving both albumin and immunoglobulin” (49). IVIG supplies are limited (100) so the supply of Flebogamma could be insufficient to meet the demand for it if the AMBAR protocol receives regulatory approval and the protocol includes Flebogamma. A further concern with IVIG is that it increases serum viscosity (101), predisposing to thromboemboli, particularly in individuals who are immobile or have vascular disease (102).

Shortages of human albumin have also been reported (103), raising the question of whether recombinant human albumin (rHA) could be substituted for human albumin in AMBAR's protocol. rHA has been reported to have a safety, tolerability, and pharmacokinetic/pharmacodynamic profile similar to human albumin (104).

Additional questions about the treatment approach used in AMBAR which need to be answered include the influence of patient age, sex, and ApoE status on slowing of AD progression, the duration of slowing of cognitive and functional decline once treatment is stopped, and whether the protocol is feasible in the many AD patients who are medically frail, particularly if maintenance of a central venous catheter is required.

## Conclusions

AMBAR's findings are encouraging, despite the questions they raise. A further study offers Grifols the opportunity to address these issues, and to optimize the protocol.

## Author Contributions

DL wrote and revised the manuscript.

## Conflict of Interest

The author declares that the research was conducted in the absence of any commercial or financial relationships that could be construed as a potential conflict of interest.
